# Asbestos ban policies and mesothelioma mortality in Greece

**DOI:** 10.1186/s12889-024-18030-x

**Published:** 2024-04-26

**Authors:** Evdoxia Gogou, Chryssi Hatzoglou, Dimitra Siachpazidou, Sotirios G. Zarogiannis, Konstantinos I. Gourgoulianis

**Affiliations:** 1 Department of Physiology, Faculty of Medicine, School of Health Sciences, University of Thessaly, BIOPOLIS, 41500 Larissa, Greece; 2 Department of Respiratory Medicine, Faculty of Medicine, School of Health Sciences, University of Thessaly, BIOPOLIS, 41110 Larissa, Greece

**Keywords:** Mesothelioma in Greece, Mortality rate, Public awareness, Epidemiology, Asbestos-ban legislation

## Abstract

**Background:**

Malignant mesothelioma is a rare form of cancer that mostly affects the pleura and has a strong link to asbestos exposure. Greece banned the use of asbestos in 2005, however, the public was already aware of this substance in the 1980s. This research aims to present an overview of Greece’s mesothelioma age-standardized mortality rates (ASMR) from 1983 to 2019 by age, gender, and geographic region and to determine whether the actions to ban asbestos impacted these rates.

**Methods:**

Data were retrieved by the Hellenic Statistical Authority (HSA) from death certificates that mentioned mesothelioma as the cause of death from 1983 to 2019 with details on the residence, gender, and age. Statistical analysis was performed using PRISM 6.0 software, a two-way ANOVA test, Trend analysis was conducted using Joinpoint Regression Program 5.0 software. The linear and non-linear model was used to calculate the age-standardized rates of annual percentage change (APC) and its 95% confidential interval (95% CI).

**Results:**

From 1983 to 2019, 850 total mesothelioma deaths were recorded, the majority of whom were males (634). A rate of 74.6% accounts for males and 25.4% for females, and the ratio of Males: Females was 3:1. Males’ ASMR and the whole population’s ASMR reached their highest levels in 2011 (0.93/100000person-years and 0.53/100000person-years, respectively). To look for potential changes between the first two decades of the 21st century, we compared the mean ASMR of each geographic region in Greece between two different 10-year subperiods (2000–2009 and 2010–2019). Except for Epirus, all regions of Greece had elevated regional ASMRs, particularly in those with the highest asbestos deposits. Notably, the ASMR in Epirus decreased from 0.54/100000person-years (2000–2009) to 0.31/100000person-years (2010–2019). After 2011, the ASMR for men and the general population stabilized. This stability is important since mesothelioma in men is associated with occupational asbestos exposure. The intriguing discovery of a lower ASMR in Epirus emphasizes the need to raise awareness of the condition and implement effective public health measures.

**Conclusions:**

In Greece, the annual ASMR for males and the whole population reached its highest level in 2011, which is positive and encouraging and may be a sign that the rate will stabilize during the following years. Moreover, this study showed that the actions made in the 1980s regarding public awareness and surveillance directly impacted the decrease in Epirus rates. Future research, continual awareness, information, and recording are needed to monitor the mesothelioma epidemic. The possible benefit of a mesothelioma registry and the epidemiological surveillance of asbestos-related diseases, particularly mesothelioma mortality, need to be addressed.

**Trial registration:**

Not applicable.

**Supplementary Information:**

The online version contains supplementary material available at 10.1186/s12889-024-18030-x.

## Background

Malignant mesothelioma (MM) is a rare and aggressive form of cancer that affects the mesothelial cells of the pleura, peritoneum, pericardium, and tunica vaginalis [1–4]. The pleural location has a particularly high relationship with asbestos exposure [1, 5]. Despite widespread asbestos use bans, mesothelioma incidence is continuously rising, but it was observed a decreasing trend in industrialized countries and among younger people aged 15 to 49 years old [6, 7]. Nonetheless, despite bans, asbestos can continue to be present in some structures, such as schools or public buildings, and the construction of residential areas near previous asbestos mines, factories, or soil containing natural asbestos may still expose people to it [8, 9]. A global ban on the use and manufacturing of asbestos has not yet been put into effect; the countries that have such a ban adopted it at different times, making it difficult to predict the risk of mesothelioma in the general population in the future [6, 10–13].

Asbestos is a silicate mineral divided into two main groups: the serpentines group, which includes chrysotile (white asbestos), and the amphiboles group, which includes crocidolite (blue asbestos), amosite (brown asbestos), tremolite, actinolite, and anthophyllite [14–16]. The majority of pleural MMs in males are inflicted by occupational exposure to amphibole asbestos [17], but also previous and more recent research showed positive and significant associations between pleural cancer and mesothelioma mortality and exposure to chrysotile asbestos fibres [18, 19]. The scientific community came to a consensus in 1964 at the New York Academy of Sciences meeting on asbestos’s role as a cause of malignant mesothelioma. Presumably, for this reason, the United States banned the use of some items asbestos-containing at the beginning of the 1970s [10, 20]. Following the establishment of the Monographs program in 1970, the IARC identified asbestos as a carcinogen in 1973. It is noteworthy to mention that the IARC revised its classification of asbestos carcinogenicity three more times in 1976, 1987, and 2012. Despite these modifications, all forms of asbestos was still classified as ascertained human carcinogen with no safe threshold of exposure for asbestos [21–23]. At the end of the 1980s, a European Directive prohibited the use of asbestos in some occupational settings (in 1988, the use of crocidolite, known as blue asbestos, was outlawed in the EU), nonetheless, the EU did not outright forbid all types of asbestos usage until 2005. Still, in the early 1990s, several Member States passed national asbestos bans [24–27].

In Europe, pleural mesothelioma incidence rates are still rising, while certain countries have seen an overal decline [28, 29]. In countries where the law banned the use of asbestos was adopted early, studies have indicated a decrease in the incidence and mortality rates of MM [10, 29–31]. Research that investigated nations with high cumulative asbestos consumption (> 2 million tons) between 1933 and 2012 found an overall, distinct linear correlation between the total amount of asbestos consumed and the total number of mesothelioma deaths [32]. When comparing the incidence rates between a nation with high MPM incidence rates and one with low rates but an early asbestos ban, the risk of pleural mesothelioma in age groups under 60 seems to have decreased due to the actions taken to limit asbestos exposure [33]. A recent study showed that contrary to worldwide trends which is overall decreased, there were notable increases in age standarized mortality rate in the middle SDI(SocioDemographic Index) areas between 1990 and 1997 and 2005–2011 [34].

Previously published data about mesothelioma mortality rate (MMR) in Greece, during the period 1983–2003, has shown a remarkable 3-fold increase in the cause-specific mortality rate during the decade 1994–2003 which was 0.156/100000population, compared with the rate of 0.047/100000population in a span from 1983 to 1993 [35]. Furthermore, the area of Epirus had the highest mesothelioma mortality rate over the period 1983–2003 which was 0.38/100000population [35]. More recent epidemiological research on MMR in Greece revealed that the mortality rate for MM significantly rose during the period 2004–2015 compared to the previous period (1983–2003) except in Epirus. Particularly, in all geographical areas, the rates increased by 4 to 6 times during the most recent period except for the mortality rate in the Epirus region which was stable [36].

Greece banned all forms of asbestos in 2005; production of asbestos reached its peak in 1996 and stopped in 1999 [25]. The productive sectors were in Zidani a region in the county of Kozani which is located in Macedonia-Thraki and in Nea lampsako in Evia which is part of Sterea Ellada and is located near to Attiki [25, 37]. Metsovo is a region which is located in Epirus and is known for a high prevalence of an endemic condition known as “Metsovo lung”—calcification of the pleura—which was discovered before 1980 [38]. Metsovo is located in the Pindos ophiolite complex including serpentine bodies mainly chrysotile but also amphibole fibers like tremolite and anthophyllite. In Epirus, there is no asbestos mining [39]. Since 1969, it has been widely known that Metsovo inhabitants whitewashed their interior walls with asbestos-contaminated soil, called “luto” in the local dialect. Bazas et al. and Konstantopoulos et al. revealed the health problems associated with “luto” in the 1980s, including pleural diseases [38, 39].

Taking into consideration the long latency period of mesothelioma (which is referred to as 20–50 years), the peak of mortality rates of this disease is expected during the early decades of the 21st century [1]. The use and production of this material happened also before 1996–1999, the increase in mortality rates could have been observed also earlier.

The primary objective of the research was to present a summary of the age-standardized mortality rates (ASMR) resulting from mesothelioma in Greece between 1983 and 2019, by geographic regions, gender, and age. Additionally, epidemiological data on ASMRs for mesothelioma in total Greece and in specific geographic areas will be processed using joinpoint analysis to determine the trends, and the annual percent change (APC) of these rates. This will help determine whether the mesothelioma epidemic in Greece has peaked, reached a plateau, or is still growing.

## Methods

We used data from The Hellenic Statistical Authority (HSA), which included all death certificates having mesothelioma as the cause of death from 1983 to 2019. Descriptive data and no ICD codes were used on death certificates that were provided by HSA. The data related to all death certificates that mentioned “mesothelioma” without specifying the exact location. Thus, the data refers to all sites of mesotheliomas. Moreover, information on the residence, gender, and age was included. The HSA gave the information under strict confidentiality conditions and based on the study’s design; it was stated that no ethical committee approval was necessary. The population figures were provided by the HAS (Hellenic Statistical Authority) and corresponded to the annual population of Greece, or of geographical areas in Greece on the 30th of June each year. The European standard population by age-group and gender were used (update 2018) [40].

According to gender, age, and geographic location in Greece, ASMRs (Age standardized Mortality Rates) have been computed for each year in a span from 1983 to 2019. To make comparisons this period was divided into 3 separate sub-periods: 1983–1999, 2000–2009, and 2010–2019. Additionally, we assessed the ASMR of Mesothelioma for each geographical region in Greece for each year between 2000 and 2019. Also, this time frame was split into two separate, 10-year subperiods: 2000–2009 and 2010–2019. In order to look for any potential changes between the first two decades of the 21st century, we compared the mean ASMR of each geographic region in Greece between two different 10-year subperiods (2000–2009 and 2010–2019). The geographical distributions represented eight regions of Greece: Macedonia-Thrace, Thessaly, Epirus, West Greece, Attica, Sterea Ellada, Peloponnisos, and Creta-Islands (Additional file 1).

Trend analysis were conducted using Joinpoint Regression Program 5.0 software. Linear model was used to calculate the age-standardized rates of annual percentage change (APC) and its 95% confidential interval (95% CI). Statistical analysis was performed using the software PRISM 6.0, the graphs were made with PRISM 6.0 and MapChart logo. The ASMRs were assessed for normality with the D’Agostino & Pearson test and parametric analysis among groups was performed with the two-way ANOVA test, Mean ± CI were estimated for each period and a *p*-value less than 0.05 was considered statistically significant. The rates are reported per 100,000 population.

## Results

**Fig. 1:**
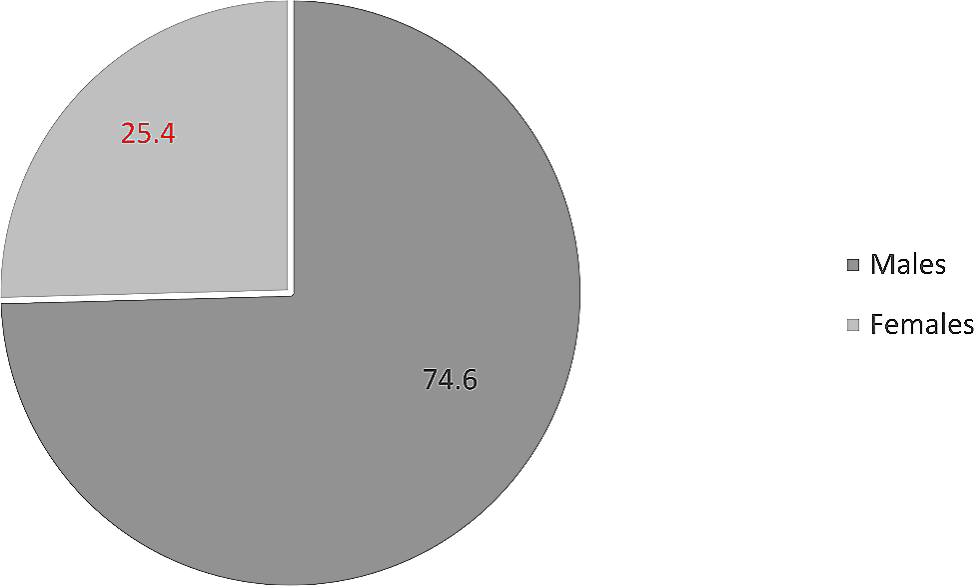
Percentages of mesothelioma deaths in Greece by gender for the period 1983–2019

In the span of 1983 to 2019 have recorded 850 total mesothelioma deaths, the majority of them 634 were males. A rate of 74.6% accounts for males and 25.4% for females, and the ratio of Males: Females was 3:1 (Fig. 1).

**Fig. 2:**
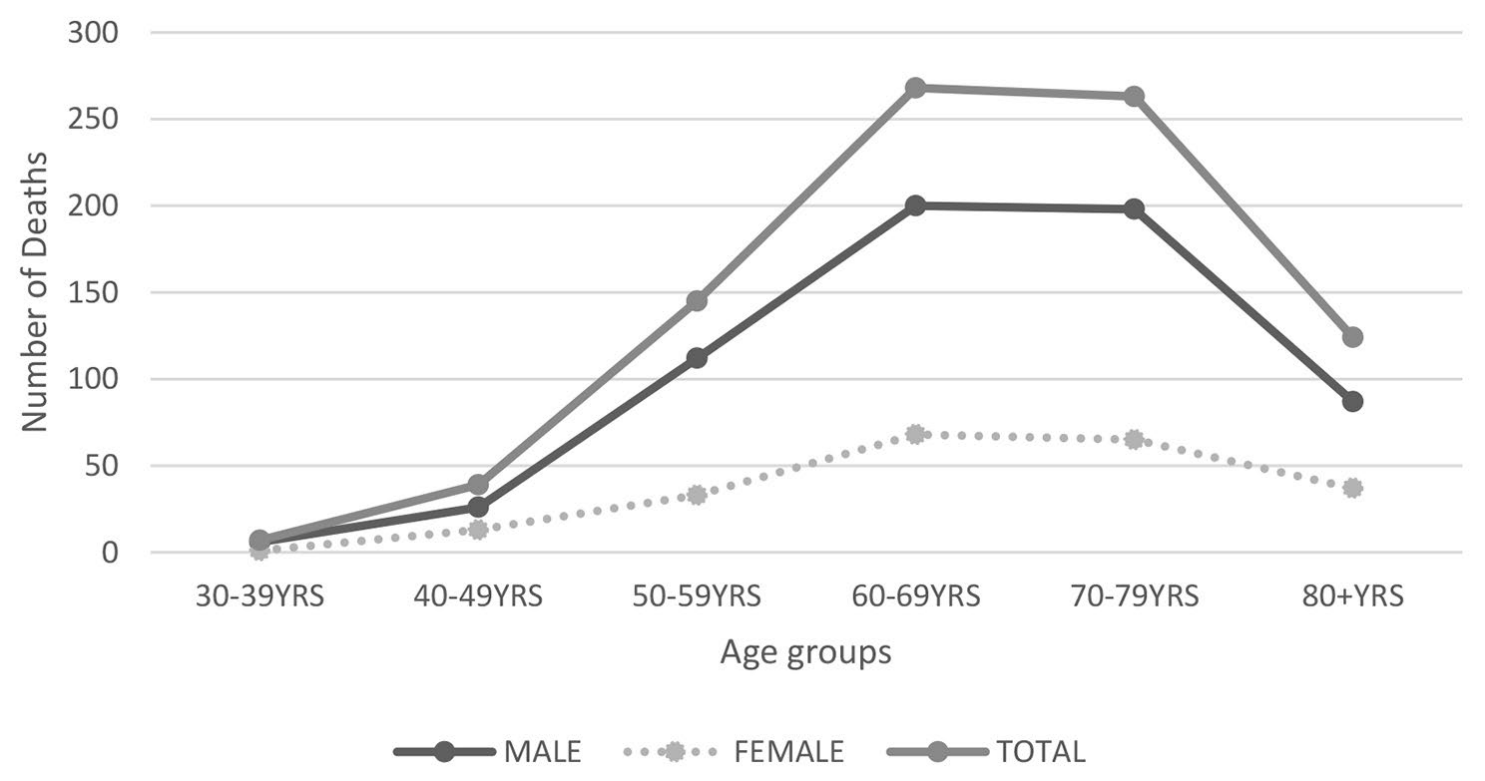
Number of mesothelioma deaths with respect to age and gender for the period 1983 to 2019

The majority of MM deaths, as shown in Fig. 2, occur in people in the age group of 60–79.

### Mesothelioma ASMR’s temporal trends

**Fig. 3:**
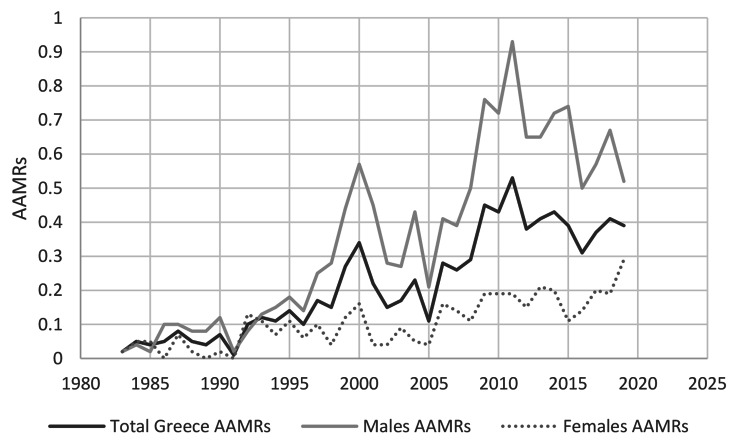
ASMR in Greece annually from 1983 to 2019 according to gender. The rates are reported per 100,000 population

Figure 3 shows the Mesothelioma Age Standardized Mortality Rate (ASMR) in males, females, and general population annually from 1983 to 2019. Both genders showed an increasing trend in the ASMR. Notably, the ASMR for men (0.93/100000person-years) and the general population (0.53/100000person-years) reached their highest point in 2011, while the ASMR for women continued to rise until 2019.

**Fig. 4:**
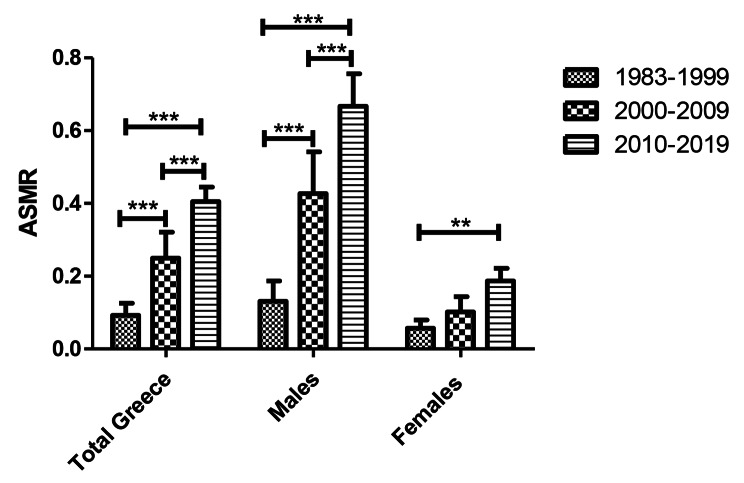
Comparison of ASMR among three consecutive subperiods 1983–1999, 2000–2009, and 2010–2019 in Greece. The rates are reported per 100.000. Two Way Anova, *** *P* < 0.001, ** *P* < 0.01, Data shown are Mean ± CI

Comparative analysis of ASMRs for the three sub-periods 1983–1999, 2000–2009, and 2010–2019 revealed a statistically significant increased trend in rates across these subsequent sub-periods. Particularly, the male’s ASMR increased gradually from 0.13/100000person-years to 0.43/100000person-years and to 0.67/100000person-years respectively during the 3 subperiods(*P* < 0.001), the female’s ASMR increased gradually from 0.05/1000000person-years to 0.10/100000person-years and to 0.19/100000person-years respectively during the 3 subperiods (*P* < 0.01 between the first and the last period), and the total population’s ASMR increased gradually from 0.09/100000person-years to 0.25/100000person-years and to 0.40/100000person-years respectively during the 3 subperiods (*P* < 0.001) (Fig. 4).

**Fig. 5:**
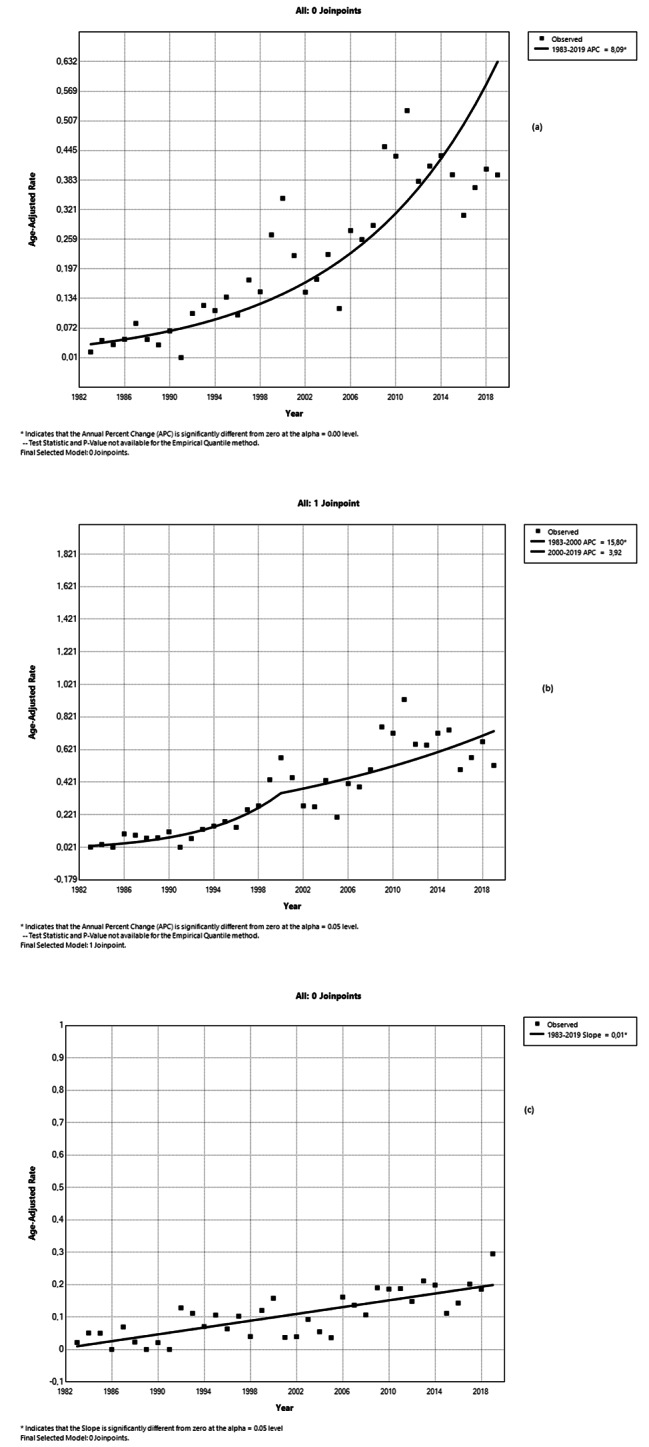
Trends of ASMRs and APC (Annual Percent Changes) in Total population **(a)**, in Males **(b)** and Females **(c)** in Greece from 1983 to 2019

Figure 5 shows the rising trends of ASMRs in males (b), females (c), and the whole population (a) from 1983 to 2019. Increased APC of the ASMRs was observed during the whole period 1983–2019. Specifically, across the whole study period, a statistically significant higher APC of 8.09 is detected for the entire population. Males had a positive APC 3.92 for the subsequent period of 2001–2019 and a much higher APC 15.08 during the period 1983–2000. Also, over the whole 1983–2019 period, the APC of female’s ASMRs is at a significant level of 0.01.

### Mesothelioma ASMR’s trends by geographical distribution

**Fig. 6:**
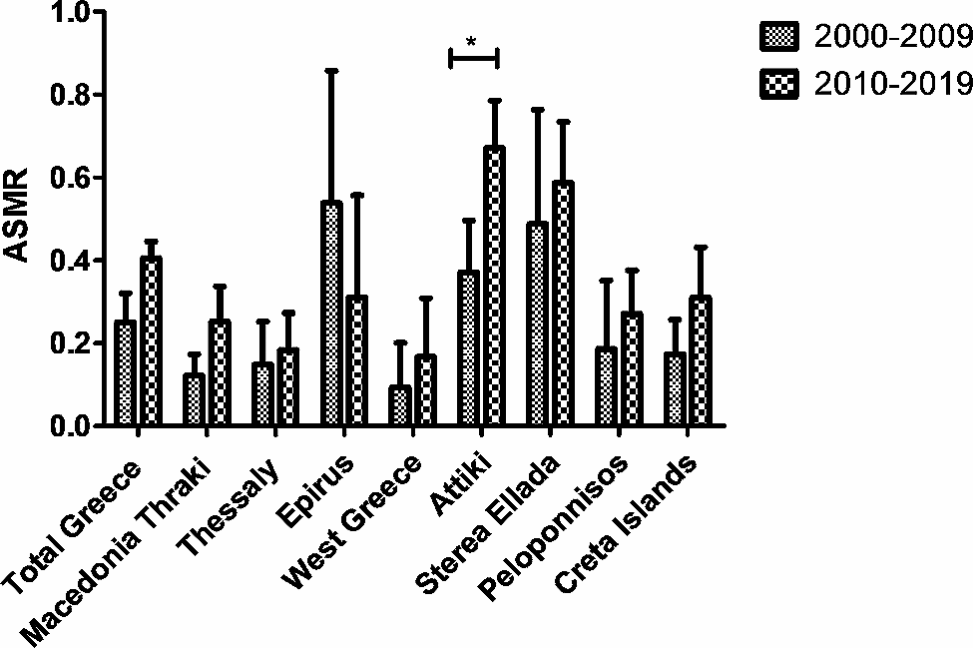
Comparison of ASMR in geographical areas in Greece between two consecutive periods 2000–2009, and 2010–2019. The rates are reported per 100.000. Two Way Anova, * *P* < 0.05. Data shown are Mean ± CI

The area-specific ASMR was calculated for each geographical region of Greece annually from 2000 to 2019. Then, comparisons of the ASMRs were made between the two consecutive periods of 2000–2009 and 2010–2019 in order to identify any differences (Fig. 6). The highest ASMR was recorded in Epirus during the first period (2000–2009), and only this ASMR decreased during the most recent period (2010–2019). In the period 2000–2009, Epirus had the highest ASMR (0.54/100000person-years), followed by Sterea Ellada (0.49/100000person-years), Attica (0.37/100000person-years), Peloponnisos (0.19/100000person-years), Crete with the islands (0.17/100000person-years), Thessaly (0.14/100000person-years), Macedonia-Thrace (0.12/100000person-years), and Western Greece (0.09/100000person-years). During the first decade 2000–2009, above the ASMR in total Greece (0.25/100,000 person-years) were observed in Epirus, Sterea Ellada and Attiki.

Attiki (0.67/100000person-years) experienced a statistically significantly more than two-fold increase in the ASMR during the most recent period of 2010–2019 (CI 0.03–0.56, *P* > 0.05), while all other regions, except Epirus, experienced an increase without statistical significance. During the second decade 2010–2019, above the ASMR in total Greece (0.40/100,000 person-years) were observed in Attiki 0.65/100000person-years, and Sterea Ellada 0.59/100000person-years. The ASMR from the other areas was increased but lower than the ASMR in the total population in Greece. Specifically, the ASMR of Crete-Islands was 0.31/100000person-years, Peloponnisos’s ASMR was 0.27/100000person-years, Macedonia-Thraki’s ASMR was (0.25/100000person-years), Thessaly’s ASMR was 0.18/100,000 person-years, and Western Greece’s ASMR was 0.17/100000person-years. The only decreased ASMR was recorded in Epirus which dropped from 0.54/100000person-years to 0.31/100000person-years in the recent period 2010–2019 but with no statistically significant difference (Fig. 6).

**Fig. 7:**
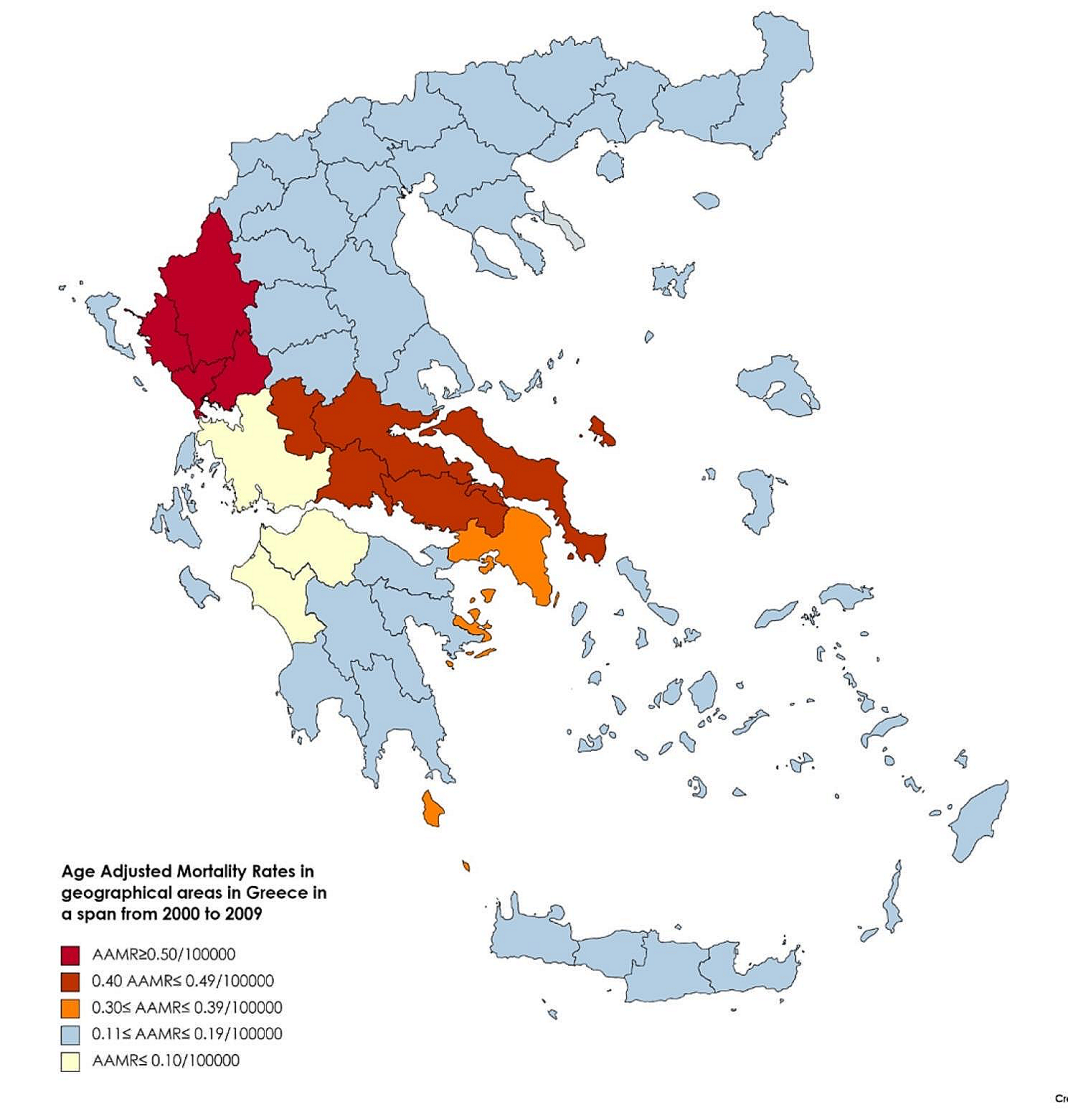
ASMR in geographical areas in Greece, regarding the total population, for the period 2000 to 2009

Figure 7 displays the mean ASMR by geographic region in Greece from 2000 to 2009 using color gradients. The red color depicts the ASMRs ≥ 0.50/100000person-years recorded in Epirus (part of West Greece, 0.54/100000person-years) and the brown color corresponds to the ASMR of Sterea Ellada (part of Central Greece, 0.49/100,000 person-years). The yellow color presents the ASMR of Attiki (a county of Sterea Ellada 0.37/100000person-years, which is above the ASMR in the whole of Greece (0.25/100000person-years) during the period 2000–2009. We display Attiki separately from Sterea Ellada since it includes Athens, the capital of Greece, where over half of the country’s population resides. The light blue color depicts ASMRs with values 0.11–0.20 below the ASMR in the total population of Greece (0.25/100000person-years) during the period 2000–2009, which were recorded in Peloponnisos (0.19/100000person-years), in Creta-Islands (0.17/100000person-years), in Thessaly (which is part of Central Greece, 0.14/100000person-years), in Macedonia-Thraki (corresponds the North part of Greece, 0.12/100000person-years). The very light color depicts ASMRs with values ≤ 0.10 which were recorded in West Creece (which includes the western part of Central Greece and the western part of Peloponnisos, 0.09/100000person-years).

**Fig. 8:**
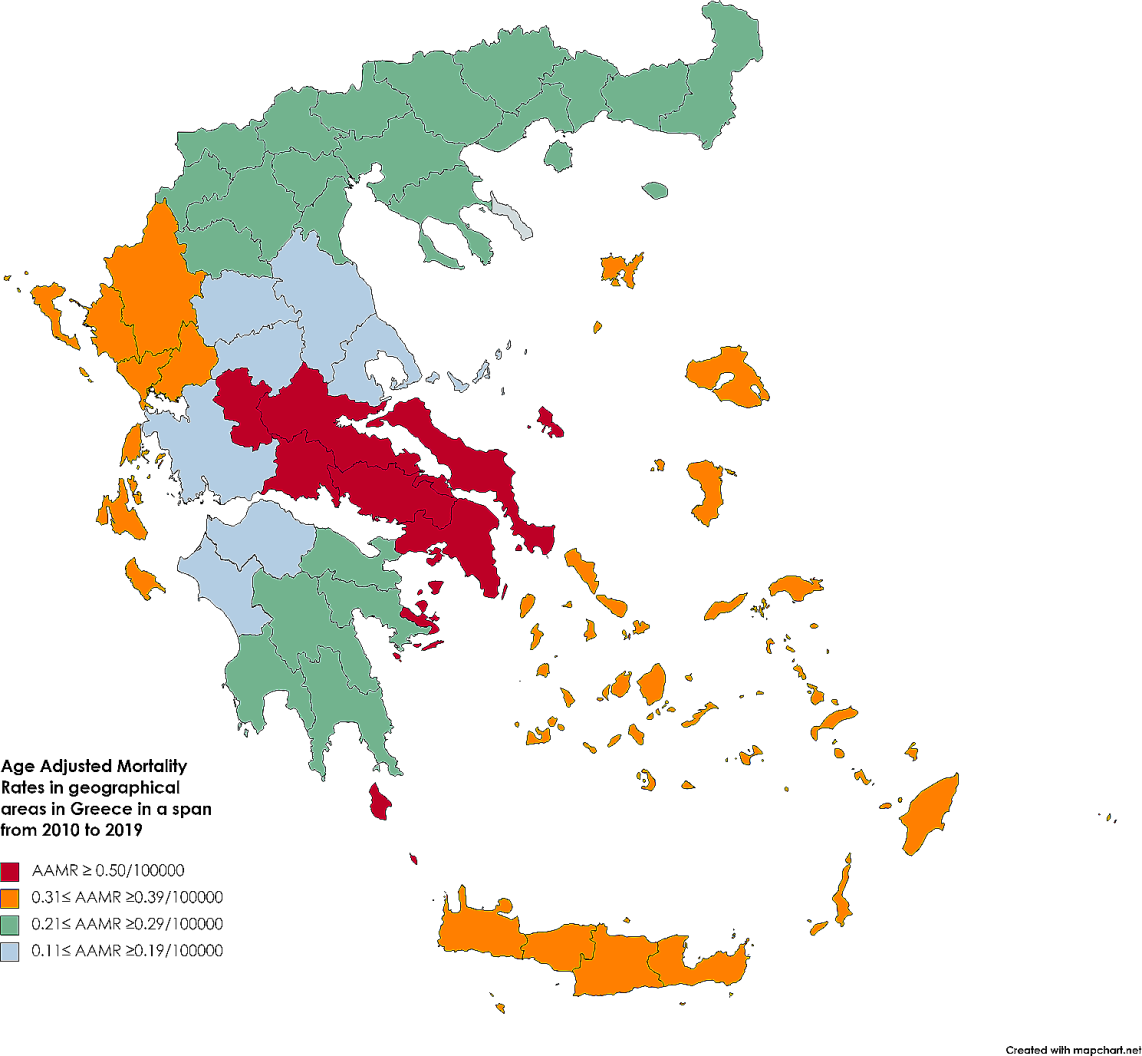
ASMR in geographical areas in Greece, regarding the total population, for the period 2010 to 2019

Figure 8 displays the mean ASMR by geographical areas of Greece for the period 2010 to 2019, using color gradients. ASMR ≥ 0.50/100000person-years was recorded in Attiki and Sterea Ellada (0.67/100000person-years, 0.59/100000person-years, respectively) The ASMR = 0.31/100,000 person-years in Epirus below the ASMR in the whole of Greece (0.40/100000person-years). The red color depicts the ASMRs ≥ 0.50/100000person-years recorded in Attiki and Sterea Ellada. The yellow color corresponds to the ASMR with values 0.31–0.39/100000person-years which was below the ASMR in the total population of Greece (0.40/100000person-years). The above values were recorded in Epirus (0.31/100000person-years) and in Creta-Islands (0.29/100000person-years). The light green color presents the ASMR in Peloponnisos (0.27/100000person-years) and in Macedonia-Thraki (0.25/100000person-years). The light blue color depicts ASMRs with values 0.11–0.20/100000person-years which were recorded in Thessaly (0.18/100000person-years), in West Greece (0.17/100000person-years).

## Discussion

Our findings confirm that men are considerably more affected by mesothelioma than women, with a male:female ratio of 3:1. The WHO mortality database, which included data on mesothelioma deaths from 1994 to 2008, also found a roughly similar ratio [26]. This ratio supports the notion that the disease primarily affects men because is caused by occupational asbestos exposure [41]. Environmental asbestos exposure has a similar impact on both genders and the ratio of male to female will not change noticeably. The male-to-female ratio varies with different types of exposure, mainly occupational. Areas with occupational sectors where workers are exposed to asbestos may have a higher male-to-female ratio, if these workers were primarily men. Women may have a higher prevalence of familial asbestos exposure (when a member of their family has occupational exposure to asbestos) [42–45]. A study suggested that the risk of MM in females is comparable to that of males, and more thorough exposure histories for females are required [46].

According to our study, mesothelioma typically affects adults within the age group of 60 and 79. This finding is supported by other studies and is compatible with the lengthy latent period of the disease [31, 47]. Mazureck et al. suggested that the majority of MM deaths, between 1999 and 2015 in the USA, occurred in both sexes between the ages of 75 and 84 [48]. A study conducted between 2005 and 2015 in southern Italy, where there was environmental exposure to ophiolite, revealed that the average age of MM deaths ranged from 65 to 77 years [42]. Data from the WHO mortality database, covering the period from 1994 to 2008, showed the average age of mesothelioma deaths was 70 years old [49].

Regardless of the gender, mesothelioma mortality rates in Greece have been rising from 1983 to 2019. This period was divided into three sub-periods 1983–1999, 2000–2009, and 2010–2019, in order to revealing the trend of MM deaths. The age-standardized mortality rates for males, and the general population doubled over the past two sub-periods, and there was a substantial increase among the three sub-periods (*p* < 0.001). The age-standardized mortality rates for females increased among the first and third sub-period (*p* < 0.01). The joinpoint analysis supported a slight but persistent rise in Females’s as well as a significant progressive increase in males’ and the overall population’s confirmed ASMRs. This increased ASMR is a result of asbestos use and production in Greece over the 20th century. Asbestos production in Greece began in the 1960s, and it grew from 1970 onward, reaching a peak in the 1990s Greek asbestos manufacturing peaked in 1996 and ceased in 1999, while asbestos exports continued until 2003 [25]. Domestic use of asbestos in Greece accounted for 20–25% of production in 1980, and a significant asbestos consumption rate of 1.9 kg/person was recorded in 1986 [25, 28]. By 1993, 95% of the fiber had been exported, and in 2005 Greece passed legislation forbidding the usage of all types of asbestos [10, 25, 28]. Based on the above data, we can conclude that there was significant asbestos production and consumption in Greece up to the mid-1990s. Given the long latent period of mesothelioma, an increase in the number of deaths from mesothelioma and consequently the ASMR in Greece during the early decades of the 21st century is anticipated. As the latent period of mesothelioma and their relation with the incidence of mesothelioma is concerned, there were several studies referred to a minimum latent period of 10 years, and other cohort studies with a maximum follow-up of 40 years after the initial asbestos exposure showed that the incidence of MM increased continuously over time after exposure, with no upper limit [50–54]. However, more recent cohort studies, that include longer periods of observation (for 50 years or longer), showed an attenuation of the pleural MM risk increase and a continuing increase for peritoneal MM [55]. Additionally, incidence trend analysis revealed that the rate of growth in incidence is reducing, especially in the countries that implemented earlier asbestos bans or reduction programs. This change is more pronounced in younger age groups exposed to lower levels of asbestos [28].

Except for the use and production of asbestos in Greece, the advancements in diagnostic techniques may have contributed to the rise in ASMRs between the three study periods (1983–1999, 2000–2009, and 2010–2019). Since mesothelioma knowledge and diagnosis have improved, there may be an association between the rising mortality rates and the increased incidence reports. The rule banning all forms of asbestos was just put into effect a short time ago in Greece, therefore it’s not surprising that ASMR has continued to rise.

The incidence and mortality mesothelioma rates are consistent with trends in asbestos exposure due to the strong causal link between asbestos and mesothelioma [28]. Studies have revealed that the time of the law’s implementation prohibiting the use of asbestos has an impact on these rates as well [10, 29–31]. MM incidence trends differ between European nations. The peak incidence of mesothelioma in Finland was reported to have occurred in 2006, and this country was one of the first in Europe to implement a law outlawing the use of asbestos early in 1992 [31]. Moreover, Sweden was among the first nations in the world where the incidence of MPM began to decline very early in 1995 [31]. Although MM mortality in the Netherlands was predicted to peak around 2020, a recent analysis suggested that MM incidence peaked earlier in 2010 [29, 56, 57]. The Netherlands was one of the countries with high rates of MM and an early national asbestos ban in 1993 [29]. Despite the early implementation of the asbestos ban law in Italy in 1992, a recent study predicts that the incidence of ARDs(Asbestos Related Diseases) will reach its peak in 2021 [28, 58]. This was attributed to the prolonged latent period of ARDs, as well as exposure to asbestos-containing products and/or environmental exposure brought on by living near sources of asbestos-like natural fibers or polluted locations [58, 59].

During the 1970s, there has been a significant decrease in occupational asbestos exposure in the USA, which resulted in a consistent decrease in MPM (Malignant Pleural Mesothelioma) incidence after 2000. Particularly, the drop was significantly higher in males than in females, with the highest MPM incidence being found in 2000 and the lowest in 2016 [30]. Australia had the largest per-capita asbestos usage, which peaked in the 1970s, and a complete ban on asbestos in 2003 [60]. The incidence rates of MM in Australia have stabilized over the first decade of the 21st century (2.8 per 100,000 in 2011) [60]. According to a research, the crude mesothelioma mortality rate has been rising in Brazil for both males and females and it’s important to mention that Brazil was one of the top four asbestos producers in 2020 [61].

Regardless of gender, the annual ASMR progressively increased from 1983 to 2019 in Greece. A closer study revealed that the rates for males and the whole population had peaked in 2011, but the ASMRs for females were still rising until 2019. The ASMRs for males and the total population were high but constant over the second decade of the 21st century, remaining below the values they had in 2011 (0.93/100000person-years and 0.53/100000person-years, respectively). These rates stabilized about 15 years after the peak of asbestos production and use in Greece, which was around in the middle of the 1990s. This stability is concerning for male’s ASMRs since the incidence of mesothelioma in men is associated with occupational asbestos exposure. In many countries, where the ASMRs for males have decreased or stabilized, the ASMR for females has continued to rise or remained more stable [10, 30, 48]. The data that is available now supports the theory that elevated female mortality rates are mostly caused by non-occupational exposure, particularly family exposure (secondary exposure when a family member is exposed to asbestos at work), which causes MM in women [62]. Additionally, it has been documented that mesothelioma is causally connected with germline mutations of BRCA1-associated protein 1 (BAP1) and various tumour suppressor genes [63]. Mesotheliomas associated with inherited germline mutations have a M:F ratio that is about 1:1 and manifest at a younger age similar to those resulting from environmental exposure [63–68]. Recent research on survival rates revealed that individuals with germline BAP1 mutations and women with malignant pleural mesothelioma have better survival rates than males [69, 70]. In terms of genetic susceptibility, MM is often diagnosed three or more decades after the asbestos exposure first occurs, but the precise genetic and molecular changes that occur during this time are still unknown. Furthermore, there is controversy on the proportion of MM cases worldwide that can be linked to genetic susceptibility. According to the present study, it is extremely positive that the annual ASMR for males and the whole population has stabilized since 2011, even though the mean ASMR in the most recent studied period (2010–2019) was double that of the previous period (2000–2009) regardless of gender.

During the most recent period 2010–2019, regional ASMRs rose in all areas of Greece except for Epirus. We found that ASMRs were significantly higher in Attiki (*P* < 0.01) and a sizable increase in Sterea Ellada and Macedonia-Thraki. It is important to note that one of the major asbestos factories, which was in operation until the middle of the 1990s, was situated in Nea Lampsako in Evia, which is part of Sterea Ellada and is near to Attiki [25]. Furthermore, the greatest asbestos deposit was in Zidani, a region in the county of Kozani, which is located in Macedonia. The asbestos mining there started in the 1970s and reached its peak in 1996 [25]. The significant increase in ASMRs in regions of Attiki, Sterea-Ellada, and Macedonia-Thraki is anticipated to occur not only in the second but also in the third decade of the 21st century, as there were the main asbestos-factories in Greece operated until 1999.

The decline in ASMR in Epirus from 0.54/100000person-years (2000–2009) to 0.31/100000person-years (2010–2019) is both noteworthy and encouraging. According to our previous research, the highest MMR in Epirus occurred between 1983 and 2003, and this rate stabilized in the period 2004–2015 [36]. At this point, we would like to highlight that Metsovo is a city in the region of Epirus, where a high prevalence of an endemic condition known as “Metsovo lung”—calcification of the pleura—was discovered before 1980 [39]. Since 1969, it has been widely known that Metsovo inhabitants whitewashed their interior walls with asbestos-contaminated soil, called “luto” in the local dialect. Bazas et al. and Konstantopoulos et al. revealed the health problems associated with “luto” in the 1980s, including pleural diseases [38, 39]. Following the implementation of a health promotion strategy, the “luto” has not been used since 1985, putting an end to the epidemic of domestic exposure [71]. According to previously published data, the MMR in Epirus was the highest in Greece before 2000, then it stabilized and recently decreased as a result of the improved surveillance, and a further decline in rate is predicted for the following years [35, 36]. The intriguing discovery of a lower ASMR in Epirus emphasizes the need of raising awareness of the condition and putting effective public health measures into practice. Therefore, it is crucial to implement a Registry for the MM incident cases so as to successfully monitor MM in Greece.

### Limitations and strengths

This study is the first to provide data about age-standardized mortality rates (ASMRs) on mesothelioma in Greece over an extensive period, from 1983 to 2019. Furthermore, the joinpoint analysis showed the trends of ASMRs and the annual percent change (APC). These could help to determine whether the manufacture, consumption, and prohibition of asbestos have had an impact on the incidence of mesothelioma in Greece. Given the consistency of the data (ASMR and APC), it is a systematic record that may be utilized in future mesothelioma epidemiological investigations. These national statistics could be used to estimate the asbestos’s impact on global health and forward evidence for a worldwide ban.

The HSA provided death certificates with descriptive data instead of ICD codes. The information pertained to every death certificate mentioning “mesothelioma” that did not provide a precise location. As a result, the data covers all mesothelioma locations. Moreover, before 1990, the HAS’s methods for recording were less advanced, therefore it’s possible that some mesothelioma deaths were missed. The possible under-reporting of MM in the death certificates should also be mentioned as a potential limitation of our study.

## Conclusions

In Greece, the annual age-standardized mesothelioma mortality rate for males and the whole population reached its highest level in 2011, which is positive and encouraging and may be a sign that the rate will stabilize during the following years. Moreover, this study showed that the actions made in the 1980s regarding public awareness and surveillance directly impacted the decrease in Epirus rates. These rates in Greece are expected to steady in the future before decreasing.

The decrease and stability of ASMRs in countries that passed asbestos ban laws early indicated the effectiveness of the measures. The possible benefit of a mesothelioma registry and the epidemiological surveillance of asbestos-related diseases, in particular mesothelioma mortality, need to be addressed. Therefore, ongoing awareness, knowledge, monitoring, and future epidemiological investigations are required to keep track of the mesothelioma epidemic.

### Electronic supplementary material

Below is the link to the electronic supplementary material.

Supplementary Material 1

## Data Availability

The data supporting this study’s findings are available on request from the corresponding author EG. The data are not publicly available due to restrictions e.g. their containing information that could compromise the privacy of research participants.

## Abbreviations

ADRs: Asbestos Related Diseases
ASMR: Age Standardized Mortality Rate
HSA: Hellenic Statistical Authority
MM: Malignant Mesothelioma
