# Supplementary figures and images for: Asbestos ban policies and mesothelioma mortality in Greece
Source: BMC Public Health. 2024 Apr 26;24:1177. doi: 10.1186/s12889-024-18030-x (PMC11055379; doi:10.1186/s12889-024-18030-x)

**Additional file 1**

**Geographical areas in Greece**


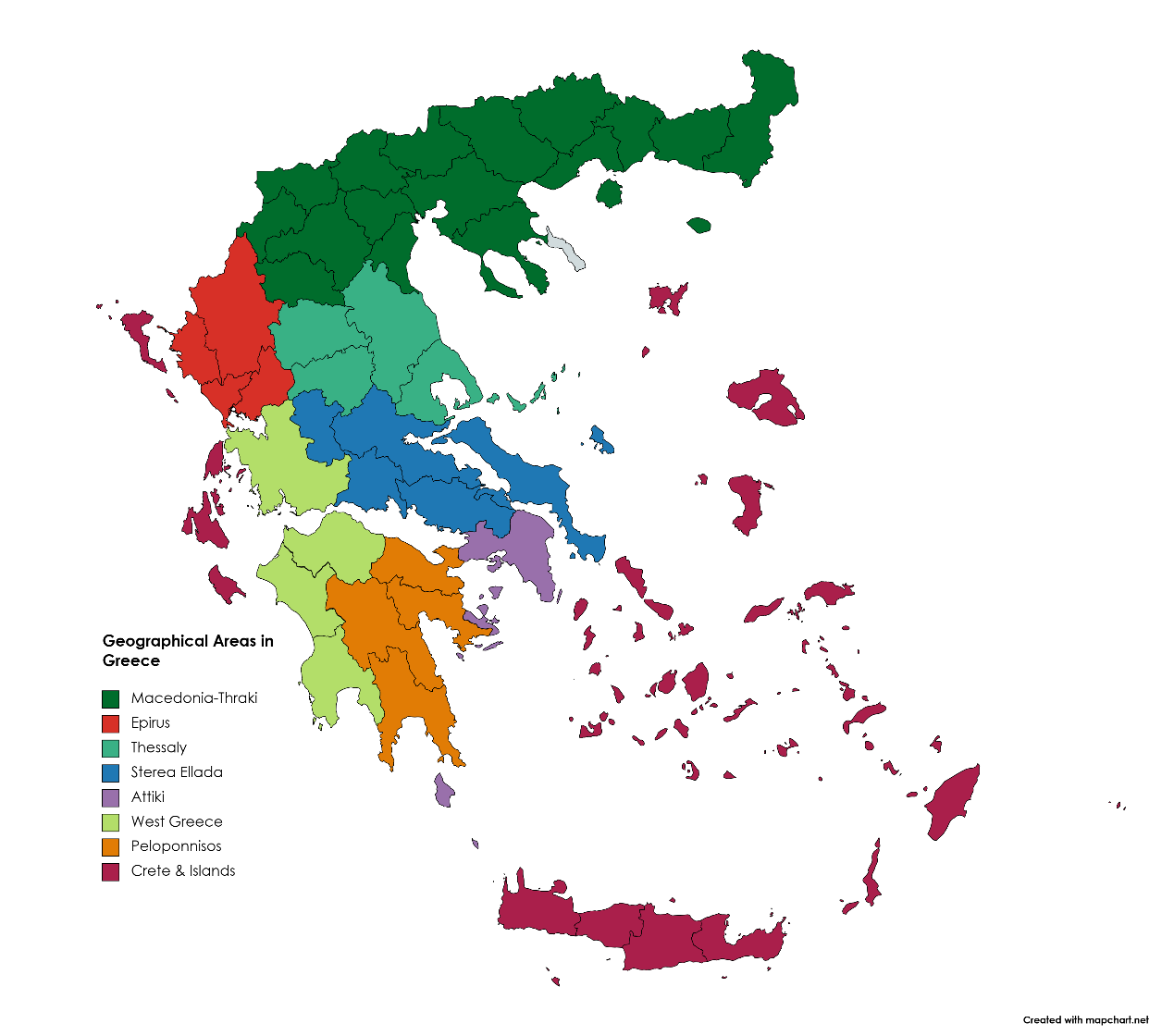

Supplement: Supplementary file 1 — Supplementary Material 1 [file 12889_2024_18030_MOESM1_ESM.docx]
